# Risk factors associated with higher skin age relative to chronological age in community-dwelling middle-aged Japanese adults

**DOI:** 10.1038/s41598-025-29647-2

**Published:** 2025-11-23

**Authors:** Toshiharu Ninomiya, Emi Oishi, Asahi Kato, Ei Teshima, Junichi Taira, Mao Shibata, Emi Ueda, Yoshihiko Furuta, Naonori Uchida, Atsushi Yamada, Naoto Minagawa, Tsuyoshi Matsuoka, Taku Otani, Kazuomi Chida, Satoko Sakata

**Affiliations:** 1 Department of Epidemiology and Public Health, Graduate School of Medical Sciences, Kyushu University, 3-1-1 Maidashi, Higashi-ku, Fukouka, 812-8582 Japan; 2 Center for Cohort Studies, Graduate School of Medical Sciences, Kyushu University, Fukuoka, Japan; 3 Department of Cardiovascular Medicine, Graduate School of Medical Sciences, Kyushu University, Fukuoka, Japan; 4 Department of Orthopaedic Surgery, Graduate School of Medical Sciences, Kyushu University, Fukuoka, Japan; 5 Department of Urology, Graduate School of Medical Sciences, Kyushu University, Fukuoka, Japan; 6 Department of Ophthalmology, Graduate School of Medical Sciences, Kyushu University, Fukuoka, Japan; 7 Department of Medicine and Clinical Science, Graduate School of Medical Sciences, Kyushu University, Fukuoka, Japan; 8 Q’sai Co., Ltd, Fukuoka, Japan; 9 HuBit genomix,Inc, Tokyo, Japan; 10 Daiko Advertising Inc, Osaka, Japan

**Keywords:** Cardiology, Diseases, Health care, Medical research, Risk factors

## Abstract

Skin condition reflects both chronological aging and overall health, yet few studies have examined its links to lifestyle or cardiovascular risk factors in Asian populations. In 2023, 527 Japanese adults aged 40–64 years underwent facial imaging with the VISIA Evolution, which estimated skin age and quantified eight skin parameters. The difference between skin and chronological age, derived from linear regression residuals, was analyzed for associations with lifestyle and cardiovascular factors using multiple regression. A greater skin–chronological age difference was significantly associated with higher scores on all eight skin parameters. In multivariable-adjusted models, male sex, higher systolic blood pressure, elevated γ-GTP, smoking, reduced grip strength, and frequent occupational sun exposure were associated with greater skin–chronological age difference. Sex-specific multiple regression identified systolic blood pressure, blood glucose, smoking, reduced grip strength, and sun exposure in women, and systolic blood pressure, serum γ-GTP, alcohol intake, reduced grip strength, and sun exposure in men. The present cross-sectional analysis showed that cardiovascular and metabolic health, as well as smoking and sunlight exposure, were associated with skin aging objectively assessed by facial imaging. Although causality cannot be inferred, improving systemic health may help promote healthier skin aging.

**Supplementary Information:**

The online version contains supplementary material available at 10.1038/s41598-025-29647-2.

## Introduction

The skin serves as a mirror reflecting the overall health of the body, and its condition is influenced by a variety of factors including environmental, physiological, and genetic components^1^. Improvement in skin condition may not only enhance quality of life and well-being, but also serve as a useful indicator for assessing general health^2^. Emerging evidence suggests that skin condition may be affected by general health status^3–6^. A recent systematic review reported that seven risk factors—aging, male gender, ethnicity, air pollution, unhealthy diets, smoking habits, and sun exposure—were associated with skin aging^6^. However, few clinical and epidemiological studies have investigated the association between skin condition and lifestyle and cardiovascular risk factors in Asian populations.

The VISIA Evolution (Canfield Scientific, Parsippany, NJ, USA) is a noninvasive high-resolution facial imaging system that captures detailed images of the skin, providing objective data for clinical assessment and monitoring^7,8^. This system estimates the absolute scores for eight skin parameters—pigmented spots, wrinkles, skin texture, pores, ultraviolet spots, brown spots, red areas, and porphyrins—and evaluates skin age as a comprehensive indicator of skin condition based on these parameters^9,10^.

The Hisayama Study is a community-based cohort study of cardiovascular and lifestyle-related diseases, which has been ongoing since 1961 in the town of Hisayama, a suburban community of the Fukuoka metropolitan area of Kyushu Island in Japan^11^. As part of this study, an epidemiological sub-study was conducted in 2023 to examine the association between lifestyle and cardiovascular risk factors and a more positive skin–chronological age difference in community-dwelling middle-aged Japanese adults.

## Methods

### Study participants

In the town of Hisayama, health checkups for lifestyle-related disease have been repeated annually since 1961^11^. In the health check-up conducted in June to September 2023, information on a sub-survey investigating factors affecting skin condition was provided both orally and in writing to residents aged 40–64 years who participated in the health checkup. Recruitment for the skin-related study was carried out in October 2023 using a web-based system. The skin-related assessment was conducted in November 2023 among 532 residents who voluntarily participated, of whom 527 individuals successfully underwent the assessment of skin age using the VISIA Evolution.

The present study was conducted in accordance with the provisions of the Declaration of Helsinki and the Ethical Guidelines for Medical and Biological Research Involving Human Subjects in Japan. The study protocol was approved by the approval of the Kyushu University Institutional Board of Clinical Research (approval no. 23158). Written informed consent was obtained from all participants.

### Skin assessment

Skin condition was assessed using the VISIA Evolution^12^. Before the skin assessment, all participants, regardless of whether they were wearing makeup, were instructed to wipe their face with a makeup remover (Bifesta Micellar Cleansing Sheet by Mandom Corporation, Osaka, Japan) and then to wipe off any residue with a cleansing cotton pad. In addition, they were asked to rest in the assessment room for at least 30 min to acclimate to the room’s temperature and humidity. The room temperature was controlled at 20–25 °C using an air conditioner and the humidity was maintained at 40%–60% using a humidifier^13^. In the VISIA Evolution, eight parameters including pigmented spots, wrinkles, skin texture, pore size, ultraviolet spots, brown spots, redness, and porphyrins, were evaluated to clarify comprehensively assess skin condition, and consequently skin aging. For analysis, the average value of the absolute score for each parameter from the left and right cheeks was used.

### Lifestyle and cardiovascular risk factors

Information on smoking habits, alcohol intake, regular exercise, frequency of working under sun exposure, nocturnal awakenings, bowel movements, and skin care was obtained by a self-administered questionnaire. Trained interviewers checked the questionnaire at the examination. Smoking and drinking habits were categorized as current or non-current. Regular exercise was defined as engaging in sports or other physical exercise, including recreational walking, at least three times a week during leisure time. Frequencies of working under sun exposure, nocturnal awakenings, and bowel movements were categorized as follows: 1, rarely; 2, several times a month; 3, several times a week; 4, almost every day. Frequency of skin care was categorized as follows: 1, rarely; 2, approximately 1–2 days a week; 3, approximately 3–4 days a week; 4, approximately 5 days a week; 5, almost every day. Height and weight were measured in light clothing without shoes, and body mass index (BMI) was calculated in kilograms per squared meter. Waist circumference was measured at the umbilical level in a standing position by a trained staff member. Handgrip strength was measured using a digital strength dynamometer (GRIP-D, T.K.K.5401; Takei Scientific Instruments, Niigata, Japan) under the direction of trained medical staff. Participants were instructed to exert the maximum grip force, alternating for each hand, and the maximum value of four trials was used. Blood pressure was measured three times in a sitting position after a rest of at least 5 min, and the mean systolic and diastolic blood pressures of the three measurements were calculated. Blood glucose levels were measured by the hexokinase method (518 of the 527 participants underwent blood-glucose measurement in a fasting state, and 9 had postprandial blood sampling). Serum total cholesterol, serum high-density lipoprotein (HDL) cholesterol, and serum uric acid levels were measured enzymatically. Serum non-HDL cholesterol was calculated by subtracting serum HDL cholesterol from serum total cholesterol. Serum aspartate aminotransferase (AST), serum alanine aminotransferase (ALT), and serum gamma-glutamyl transferase (γ-GTP) were enzymatically measured in accordance with the consensus method of the Japan Society of Clinical Chemistry.

### Statistical analysis

The difference between skin age and chronological age was calculated as the residual of a linear regression model in which chronological age was the independent variable and skin age was the dependent variable. The associations of cardiovascular and lifestyle risk factors with the difference between skin and chronological age were assessed using a multiple regression analysis. Heterogeneity in the association between sexes was tested by adding a multiplicative interaction term between each factor and sex to the relevant regression model. In addition, variable selection was performed using a backward elimination procedure with a selection threshold of *p* < 0.10 to identify factors associated with the difference between skin and chronological age, where age and sex were included in the model irrespective of statistical significance. Analyses were performed using SAS version 9.4 (SAS Institute, Cary, NC, USA).

## Results

The mean age of participants was 52.0 years (SD: 7.1), and 30.7% were male. Clinical characteristics of the study participants are presented in Table 1. Among the overall study participants, 26.0% (women: 19.2%; men: 41.4%) worked under sunlight exposure several times per week to almost daily. In addition, 37.0% (women: 35.9%; men: 39.5%) experienced nighttime awakenings several times a week to almost daily, and 2.8% (women: 1.6%; men: 5.6%) experienced constipation or diarrhea. Furthermore, 65.3% (women: 83.6%; men: 24.1%) performed skin care five or more days a week.

**Table 1:** Clinical characteristics of the study participants.

Variables	Overall (*n* = 527)	Women (*n* = 365)	Men (*n* = 162)
Age, years	52.0 (7.1)	51.8 (7.2)	52.6 (7.0)
Men, %	30.7	0.0	100.0
Systolic blood pressure, mmHg	115.9 (14.6)	113.6 (14.7)	120.9 (13.2)
Diastolic blood pressure, mmHg	70.5 (11.1)	68.2 (10.5)	75.5 (10.6)
Blood glucose^b^, mmol/L	5.63 (0.76)	5.50 (0.63)	5.91 (0.93)
Hemoglobin A1c, %	5.7 (0.5)	5.7 (0.4)	5.8 (0.6)
Serum non-HDL cholesterol, mmol/L	3.73 (0.91)	3.74 (0.94)	3.70 (0.87)
Serum HDL cholesterol, mmol/L	1.78 (0.45)	1.88 (0.44)	1.56 (0.39)
Serum uric acid, µmol/L	309.4 (81.1)	279.9 (64.3)	375.9 (75.7)
Serum AST, IU/L	19.0 (16.0–23.0)	19.0 (16.0–22.0)	21.0 (18.0–27.0)
Serum ALT, IU/L	17.0 (13.0–24.0)	15.0 (12.0–21.0)	23.0 (16.0–35.0)
Serum γ-GTP, IU/L	22.0 (15.0–39.0)	18.0 (13.0–30.0)	35.0 (23.0–55.0)
Body mass index, kg/m^2^	23.4 (3.9)	23.0 (4.0)	24.5 (3.2)
Waist circumference, cm	83.4 (10.3)	82.0 (10.1)	86.6 (10.0)
Current smoking habits, %	6.8	4.7	11.7
Current alcohol drinking, %	56.7	50.8	69.8
Handgrip strength, kg	30.5 (9.2)	25.5 (4.5)	41.7 (6.8)
Regular exercise, %	40.2	36.2	49.4
Frequency of working under sunlight exposure: several times a week to almost every day, %	26.0	19.2	41.4
Frequency of nighttime awakenings: several times a week to almost every day, %	37.0	35.9	39.5
Frequency of constipation or diarrhea: several times a week to almost every day, %	2.8	1.6	5.6
Frequency of skin care: approximately 5 days a week to almost every day, %	65.3	83.6	24.1
Score for skin parameters from the VISIA Evolution^a^			
Pigmented spots	0.353 (0.092)	0.319 (0.074)	0.431 (0.079)
Wrinkles	0.365 (0.135)	0.361 (0.140)	0.372 (0.123)
Skin texture	0.132 (0.075)	0.097 (0.046)	0.209 (0.069)
Pores	0.251 (0.122)	0.230 (0.124)	0.298 (0.101)
Ultraviolet spots	0.246 (0.073)	0.265 (0.063)	0.204 (0.078)
Brown spots	0.211 (0.058)	0.211 (0.056)	0.210 (0.064)
Red areas	0.184 (0.145)	0.132 (0.088)	0.300 (0.178)
Porphyrins	0.128 (0.089)	0.093 (0.063)	0.207 (0.088)

Values are shown as mean (standard deviation), median (interquartile range), or frequency.

*HDL* high-density lipoprotein, *AST* aspartate aminotransferase, *ALT* alanine aminotransferase, *γ-GTP* gamma-glutamyl transpeptidase.

^a^The mean value of the absolute scores for the left and right cheeks was used for analysis.

^b^Among 527 participants, 518 provided blood samples in a fasting state, and 9 had postprandial blood sampling.

**Fig. 1:**
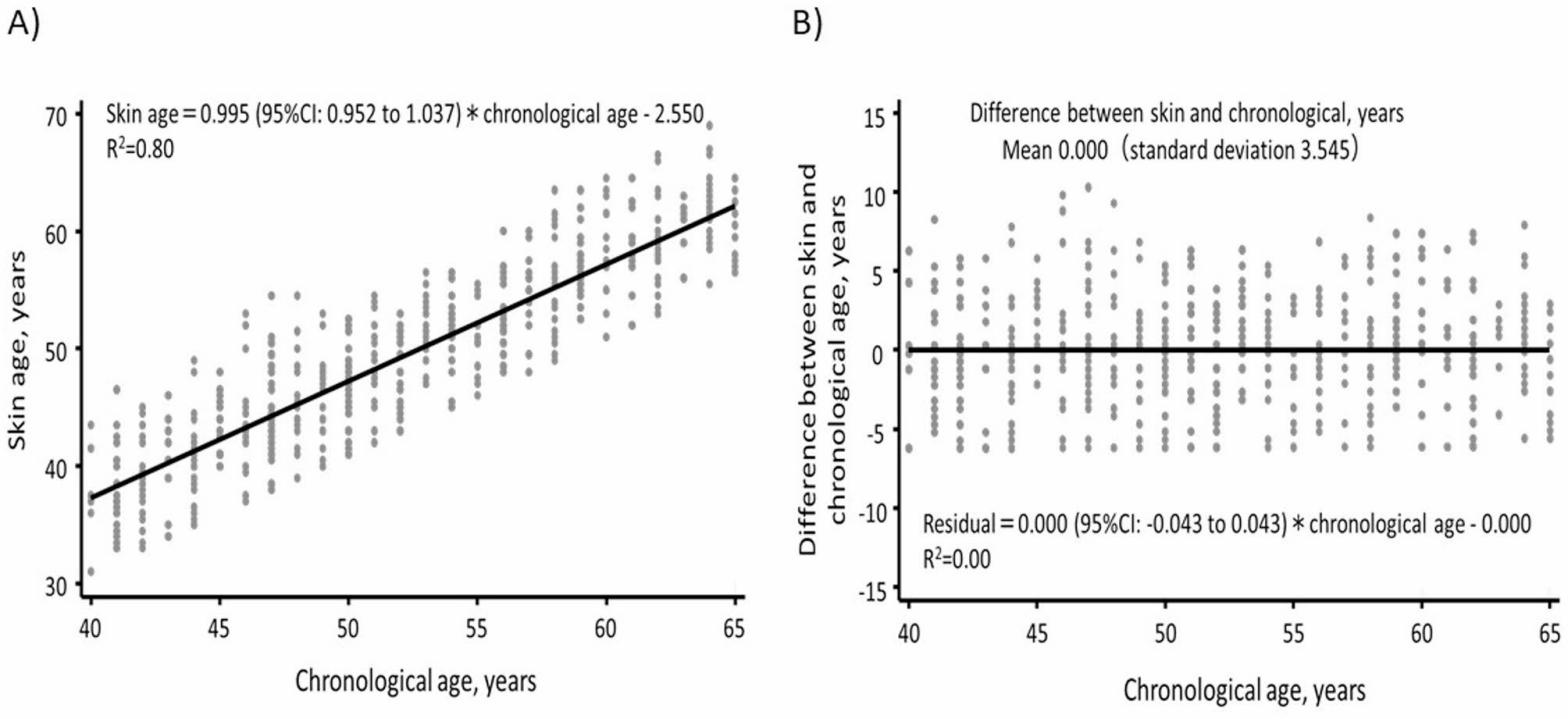
Association of chronological age with skin age (**A**) and with the difference between skin age and chronological age, calculated as the residual from a linear regression model of skin age on chronological age (**B**).

Figure 1 shows the association between chronological age and skin age, as well as the association between chronological age and the difference between skin and chronological age. A strong positive linear correlation was observed between chronological and skin age (*R*^2^ = 0.80). The mean difference between skin age and chronological age was 0.000 (SD: 3.545), and no association was found between chronological age and the skin–chronological age difference (*R*^2^ = 0.00). As shown in Table 2, greater absolute scores of each of the eight VISIA parameters—pigmented spots, wrinkles, skin texture, pores, ultraviolet spots, brown spots, red areas, and porphyrins—were significantly associated with a higher skin–chronological age difference (i.e., higher skin age relative to chronological age) after adjusting for sex and age (all *p* < 0.001). Similar significant associations were observed in both sexes, except in the case of porphyrins in women and pores and ultraviolet spots in men (Supplementary Table 1).

**Table 2:** Association of absolute scores for the parameters measured by the VISIA evolution with the difference between skin age and chronological age in the age- and sex-adjusted model.

Absolute scores for skin parameters from the VISIA Evolution^a^	Unit^b^	Overall	
		Difference (95% CI) between skin age and chronological age, years ^c)^	*p* value
Pigments spots	(per 1SD increment)	2.491 (2.178 to 2.804)	< 0.001
Wrinkles	(per 1SD increment)	1.754 (1.483 to 2.025)	< 0.001
Skin texture	(per 1SD increment)	2.838 (2.485 to 3.191)	< 0.001
Pores	(per 1SD increment)	0.725 (0.416 to 1.033)	< 0.001
Ultraviolet spots	(per 1SD increment)	0.821 (0.485 to 1.157)	< 0.001
Brown spots	(per 1SD increment)	1.725 (1.450 to 2.000)	< 0.001
Red areas	(per 1SD increment)	1.884 (1.558 to 2.209)	< 0.001
Porphyrins	(per 1SD increment)	0.531 (0.152 to 0.910)	0.006

*SD* standard deviation, *CI* confidence interval.

^a^The mean value of the absolute scores for the left and right cheeks was used for analysis.

^b^Standard deviations of each VISIA parameter were based on the data shown in Table 1.

^c^Difference between skin age and chronological age was calculated as the residual of the linear regression model of skin age on chronological age. Values were adjusted for age and sex.

With regard to the association of lifestyle and cardiovascular risk factors with the age- and sex-adjusted difference between skin and chronological age, several factors were significantly or marginally (*p* < 0.10) associated with a greater skin–chronological age difference. Specifically, higher systolic and diastolic blood pressure, blood glucose, serum uric acid, and γ-GTP levels, increased frequencies of current smoking, working under sunlight exposure, and nighttime awakenings, and lower serum HDL cholesterol levels and grip strength were associated with an increased age- and sex-adjusted difference between skin and chronological age (Table 3). In the subgroup analysis of sex, significant heterogeneity in the association between sexes was observed, with stronger associations for blood glucose and serum HDL cholesterol in women, and for serum γ-GTP and current alcohol drinking in men (Supplementary Table 2) .

**Table 3:** Association of lifestyle and cardiovascular risk factors with the difference between skin age and chronological age in the age- and sex-adjusted model.

Lifestyle and cardiovascular risk factors	Unit	Overall	
		Difference (95% CI) between skin age and chronological age, years^c^	*p* value
Systolic blood pressure	(per 10 mmHg increment)	0.406 (0.194 to 0.618)	< 0.001
Diastolic blood pressure	(per 5 mmHg increment)	0.281 (0.139 to 0.423)	< 0.001
Blood glucose^d^	(per 1 mmol/L increment)	0.422 (0.003 to 0.842)	0.048
Serum non-HDL cholesterol	(per 1 mmol/L increment)	0.158 (-0.182 to 0.498)	0.36
Serum HDL cholesterol	(per 1 mmol/L decrement)	0.712 (-0.003 to 1.427)	0.051
Serum uric acid	(per 100 µmol/L increment)	0.551 (0.101 to 1.002)	0.02
Serum AST	(per 2-times increment)	0.290 (-0.360 to 0.940)	0.38
Serum ALT	(per 2-times increment)	0.321 (-0.098 to 0.740)	0.13
Serum γ-GTP	(per 2-times increment)	0.530 (0.206 to 0.853)	0.001
Body mass index	(per 1 kg/m^2^ increment)	0.038 (-0.042 to 0.119)	0.35
Waist circumference	(per 10 cm increment)	0.130 (-0.174 to 0.434)	0.40
Current smoking habits	(yes vs. no)	1.212 (0.003 to 2.421)	0.049
Current alcohol drinking	(yes vs. no)	0.082 (-0.541 to 0.704)	0.80
Handgrip strength, kg	(per 5 kg decrement)	0.428 (0.140 to 0.717)	0.004
Regular exercise	(yes vs. no)	0.113 (-0.517 to 0.743)	0.72
Frequency of working under sunlight exposure	(per 1-category increase in questionnaire^a^)	0.315 (0.004 to 0.625)	0.047
Frequency of nighttime awakenings	(per 1-category increase in questionnaire^a^)	0.242 (-0.033 to 0.517)	0.08
Frequency of constipation or diarrhea	(per 1-category increase in questionnaire^a^)	0.161 (-0.442 to 0.764)	0.60
Frequency of skin care	(per 1-category increase in questionnaire^b^)	-0.143 (-0.359 to 0.074)	0.20

*CI* confidence interval, *HDL* high-density lipoprotein, *AST* aspartate aminotransferase, *ALT* alanine aminotransferase, *γ-GTP* gamma-glutamyl transpeptidase.

^a^Definition of category: 1, rarely; 2, several times a month; 3, several times a week; 4, almost every day.

^b^Definition of category: 1, rarely; 2, approximately 1–2 days a week; 3, approximately 3–4 days a week; 4, approximately 5 days a week; 5, almost every day.

^c^Difference between skin age and chronological age was calculated as the residual of a linear regression model with chronological age as the independent variable and skin age as the dependent variable. Values were adjusted for age and sex.

^d^Among 527 participants, 518 provided blood samples in a fasting state, and 9 had postprandial blood sampling.

Finally, multiple regression analyses were performed using full models that included factors found to be significant or marginally significant in either sex in the age- and sex-adjusted analyses (Table 4), followed by a backward elimination procedure for variable selection. In the overall population, male sex, higher systolic blood pressure and serum γ-GTP, current smoking habits, reduced grip strength, and increased frequency of working in sunlight were identified as factors associated significantly or marginally with increased difference between chronological age and skin age. In sex-specific models, systolic blood pressure, blood glucose, current smoking habits, reduced grip strength, and frequency of working in sunlight were selected in women, whereas systolic blood pressure, serum γ-GTP, current alcohol drinking, reduced grip strength, and frequency of working in sunlight were selected in men (Supplementary Table 3) .

**Table 4:** Association of lifestyle and cardiovascular risk factors with the difference between skin age and chronological age in the multivariable-adjusted model.

Lifestyle and cardiovascular risk factors	Unit	Multivariable-adjusted (Full-model)		Multivariable-adjusted (Backward elimination)^d^	
		Difference (95% CI) between skin age and chronological age, years^c^	P value	Difference (95% CI) between skin age and chronological age, years^c^	p value
Overall					
Age	(per10 years increment)	-0.423 (-0.880 to 0.035)	0.07	-0.369 (-0.801 to 0.063)	0.09
Men	(vs. Women)	0.869 (-0.425 to 2.164)	0.19	1.267 (0.116 to 2.419)	0.03
Systolic blood pressure	(per 10 mmHg increment)	0.293 (0.071 to 0.514)	0.010	0.339 (0.124 to 0.553)	0.002
Blood glucose^e^	(per 1 mmol/L increment)	0.217 (-0.231 to 0.666)	0.34	ns	
Serum non-HDL cholesterol	(per 1 mmol/L increment)	-0.087 (-0.449 to 0.274)	0.63	ns	
Serum HDL cholesterol	(per 1 mmol/L decrement)	0.601 (-0.184 to 1.387)	0.13	ns	
Serum uric acid	(per 100 µmol/L increment)	0.287 (-0.198 to 0.772)	0.25	ns	
Serum γ-GTP	(per 2-times increment)	0.447 (0.099 to 0.794)	0.01	0.490 (0.168 to 0.811)	0.003
Waist circumference	(per 10 cm increment)	-0.169 (-0.515 to 0.176)	0.34	ns	
Current smoking habits	(yes vs. no)	0.836 (-0.376 to 2.047)	0.18	0.995 (-0.183 to 2.173)	0.098
Current alcohol drinking	(yes vs. no)	0.039 (-0.607 to 0.684)	0.91	ns	
Handgrip strength, kg	(per 5 kg decrement)	0.497 (0.206 to 0.788)	< 0.001	0.478 (0.195 to 0.762)	0.001
Frequency of working under sunlight exposure	(per 1-category increase in questionnaire^a^)	0.359 (0.051 to 0.667)	0.02	0.380 (0.074 to 0.686)	0.02
Frequency of nighttime awakenings	(per 1-category increase in questionnaire^a^)	0.167 (-0.109 to 0.443)	0.23	ns	
Frequency of constipation or diarrhea	(per 1-category increase in questionnaire^a^)	-0.044 (-0.647 to 0.558)	0.89	ns	
Frequency of skin care	(per 1-category increase in questionnaire^b^)	-0.025 (-0.244 to 0.193)	0.82	ns	

CI, confidence interval; HDL, high-density lipoprotein; γ-GTP, gamma-glutamyl transpeptidase; ns, not selected.

^a^Definition of category: 1, rarely; 2, several times a month; 3, several times a week; 4, almost every day.

^b^Definition of category: 1, rarely; 2, approximately 1–2 days a week; 3, approximately 3–4 days a week; 4, approximately 5 days a week; 5, almost every day.

^c^Difference between skin age and chronological age was calculated as the residual of a linear regression model with chronological age as the independent variable and skin age as the dependent variable.

^d^Variable selection was performed using a backward elimination procedure with a selection threshold of *p* < 0.10 to identify factors associated with the difference between skin age and chronological age, where age and sex were included in the model irrespective of statistical significance.

^e^Among 527 participants, 518 provided blood samples in a fasting state, and 9 had postprandial blood sampling.

## Discussion

The present study demonstrated that several lifestyle and cardiovascular risk factors were associated with an older skin age, as objectively measured by the VISIA Evolution, relative to chronological age in community-dwelling middle-aged Japanese adults. These factors included male sex, elevated blood pressure, high blood glucose and serum γ-GTP levels, current smoking, reduced grip strength, and frequent exposure to sunlight. Notably, the subgrouping by sex showed that higher blood glucose and current smoking habits were more strongly associated with older skin age in women, whereas current alcohol drinking and higher serum γ-GTP levels showed a stronger association in men. Although causal relationships cannot be determined due to the cross-sectional nature of the study, these findings suggest that the skin aging, as measured by indices of facial skin condition, may reflect overall health status influence by lifestyle and cardiovascular factors, as well as chronological aging itself, rather than direct causal effects of these factors on skin aging.

Several previous clinical and epidemiological studies have investigated the risk factors associated with skin aging. Leung et al. reported that both smoking habits and sun exposure were significant risk factors contributing to skin aging in older adults^3^. In particular, smoking had a strong impact on increased wrinkling and visible signs of aging. Asakura et al. similarly found that smoking and failure to use sun protection were significantly associated with visible signs of skin aging in both sexes among Japanese adults aged 65 and older^4^. Clatici et al. found that skin aging was influenced by several lifestyle factors, including sun exposure, sugar intake, smoking, inadequate skin care, chronic stress, poor sleep, and intrinsic aging. Among these factors, all except intrinsic aging were largely modifiable extrinsic factors, presenting opportunities to maintain healthier and more youthful skin^5^. A meta-analysis revealed seven notable risk factors for various skin aging phenotypes—namely, chronological aging, gender, ethnicity, air pollution, nutritional status, smoking habits, and sun exposure^6^. Our present findings that smoking habits and frequent sunlight exposure were significantly associated with older skin age relative to chronological age agreed with their results. However, the present study further identified cardiovascular risk factors (i.e., elevated blood pressure, and serum γ-GTP levels) and reduced handgrip strength as risk factors associated with skin aging. Our findings suggest that, in addition to traditional extrinsic factors, skin aging may also reflect broader aspects of an individual’s general health, such as cardiovascular condition and physical function.

Several biological mechanisms may explain the risk factors found to be associated with older skin age relative to chronological age in this study. Ultraviolet radiation accelerates photoaging through DNA damage, oxidative stress, and collagen degradation^14,15^. Smoking also contributes to oxidative stress, reduced blood flow, and impaired collagen synthesis^1,5^. Elevated serum γ-GTP and uric acid levels may reflect underlying metabolic disturbances such as alcohol-induced hepatic steatosis or non-alcoholic fatty liver disease, subsequently inducing higher cardiovascular risk such as elevated blood pressure and blood glucose and dyslipidemia^16–18^. Traditional cardiovascular risk factors may contribute to chronic inflammation, glycation, endothelial dysfunction, and oxidative stress, which would likely lead to collagen degradation and impaired dermal structure^19,20^. Reduced grip strength, a marker of frailty or poor physical condition, might reflect systemic aging, which also affects the skin^21,22^. Taken together, these mechanisms suggest that skin aging may result from cumulative systemic processes, including oxidative stress, inflammation, and metabolic dysregulation.

The strengths of this study include its use of high-resolution imaging (VISIA Evolution) to objectively assess skin age, and its comprehensive measurement of lifestyle and metabolic factors among community-dwelling middle-aged adults. However, several limitations should be noted. First, the cross-sectional design excluded any inference of causality. Second, in the present study, there was insufficient information on dietary habits, supplements, use of cosmetics, and skin care practices that affect skin age. Third, the data on dietary habits, supplement use, and cosmetic or skin care practices were not collected, and that residual confounding cannot be ruled out. Finally, the generalizability of these results is limited because this study was conducted in a single region of Japan and may have been influenced by a healthy screenee bias due to voluntary participation. Therefore, these findings should be validated in other populations and ethnic groups.

## Conclusions

The present study demonstrated that factors such as male sex, elevated systolic blood pressure, increased blood glucose and serum γ-GTP levels, current smoking or alcohol consumption, reduced handgrip strength, and frequent work under sun exposure were associated with older skin age relative to chronological age, suggesting their contribution to accelerated skin aging.

These findings suggest that public health efforts, such as protecting the skin from UV exposure, preventing lifestyle-related diseases, avoiding smoking and alcohol consumption, and maintaining muscle strength through exercise, may help slow skin aging and reduce the gap between skin age and chronological age, through the overall improvement of systemic health rather than the direct effects of these individual factors. Future studies, including longitudinal analyses and studies integrating molecular markers, may provide a more comprehensive understanding of skin aging.

## Supplementary Information

Below is the link to the electronic supplementary material.

Supplementary Material 1

## Data Availability

The data set utilized in the present study is not publicly available due to its inclusion of confidential clinical data on the study participants. However, the data are available upon reasonable request and with the permission of the principal Investigator of this study, Professor Toshiharu Ninomiya.
