# Supplementary material for: Risk factors associated with higher skin age relative to chronological age in community-dwelling middle-aged Japanese adults
Source: Sci Rep. 2025 Nov 23;15:45201. doi: 10.1038/s41598-025-29647-2 (PMC12749942; doi:10.1038/s41598-025-29647-2)
Supplement: Supplementary file 1 — Supplementary Material 1 [file 41598_2025_29647_MOESM1_ESM.docx]

| **Supplementary Table 1. Association of absolute scores for the parameters measured by the VISIA Evolution with the difference between skin age and chronological age** **by sex, in the age-adjusted model** | | | | | |
| --- | --- | --- | --- | --- | --- |
| **Absolute scores for skin parameters from the VISIA Evolution ^a)^** | Unit ^b)^ | Women | | Men | |
|  |  | Difference (95% CI) between chronological age and skin age, years ^c)^ | p value | Difference (95% CI) between chronological age and skin age, years ^c)^ | p value |
| Pigmented spots | (per 1SD increment) | 2.702 (2.322 to 3.081) | <0.001 | 2.068 (1.510 to 2.626) | <0.001 |
| Wrinkles | (per 1SD increment) | 1.831 (1.517 to 2.145) | <0.001 | 1.551 (1.008 to 2.094) | <0.001 |
| Skin texture | (per 1SD increment) | 3.716 (3.232 to 4.201) | <0.001 | 1.980 (1.475 to 2.484) | <0.001 |
| Pores | (per 1SD increment) | 0.899 (0.547 to 1.250) | <0.001 | 0.126 (-0.522 to 0.775) | 0.70 |
| Ultraviolet spots | (per 1SD increment) | 1.025 (0.588 to 1.462) | <0.001 | 0.513 (-0.016 to 1.041) | 0.06 |
| Brown spots | (per 1SD increment) | 1.727 (1.376 to 2.079) | <0.001 | 1.757 (1.317 to 2.196) | <0.001 |
| Red areas | (per 1SD increment) | 2.390 (1.829 to 2.951) | <0.001 | 1.639 (1.269 to 2.009) | <0.001 |
| Porphyrins | (per 1SD increment) | 0.347 (-0.185 to 0.878) | 0.20 | 0.741 (0.210 to 1.272) | 0.007 |
| Abbreviations: SD, standard deviation; CI, confidence interval.   1. The mean value of the absolute scores for the left and right cheeks was used for analysis. 2. Standard deviations of each VISIA parameter were based on the data shown in Table 1. 3. The difference between skin age and chronological age was calculated as the residual of the linear regression model of skin age on chronological age. Values were adjusted for age. | | | | | |

| **Supplementary Table 2. Association of lifestyle and cardiovascular risk factors with the difference between skin and chronological age by sex, in the age-adjusted model** | | | | | | |
| --- | --- | --- | --- | --- | --- | --- |
| Lifestyle and cardiovascular risk factors | Unit | Women | | Men | | p for heterogeneity between sexes |
|  |  | Difference (95% CI) between chronological age and skin age, years ^c)^ | p  value | Difference (95% CI) between chronological age and skin age, years ^c)^ | p  value |  |
| Systolic blood pressure | (per 10 mmHg increment) | 0.344 (0.090 to 0.598) | 0.008 | 0.582 (0.188 to 0.976) | 0.004 | 0.32 |
| Diastolic blood pressure | (per 5 mmHg increment) | 0.294 (0.120 to 0.468) | <0.001 | 0.252 (0.002 to 0.502) | 0.046 | 0.80 |
| Blood glucose ^d)^ | (per 1 mmol/L increment) | 0.826 (0.219 to 1.434) | 0.008 | 0.043 (-0.532 to 0.617) | 0.88 | 0.02 |
| Serum non-HDL cholesterol | (per 1 mmol/L increment) | -0.018 (-0.433 to 0.397) | 0.93 | 0.602 (-0.008 to 1.211) | 0.053 | 0.10 |
| Serum HDL cholesterol | (per 1 mmol/L decrement) | 0.839 (-0.002 to 1.680) | 0.051 | 0.336 (-1.052 to 1.723) | 0.63 | 0.02 |
| Serum uric acid | (per 100 µmol/L increment) | 0.460 (-0.130 to 1.050) | 0.13 | 0.704 (0.005 to 1.402) | 0.048 | 0.27 |
| Serum AST | (per 2-times increment) | 0.261 (-0.627 to 1.149) | 0.56 | 0.359 (-0.615 to 1.333) | 0.47 | 0.85 |
| Serum ALT | (per 2-times increment) | 0.418 (-0.133 to 0.968) | 0.14 | 0.209 (-0.469 to 0.886) | 0.54 | 0.67 |
| Serum γ-GTP | (per 2-times increment) | 0.305 (-0.105 to 0.714) | 0.14 | 0.977 (0.452 to 1.501) | <0.001 | 0.05 |
| Body mass index | (per 1 kg/m^2^ increment) | 0.024 (-0.068 to 0.117) | 0.61 | 0.088 (-0.078 to 0.255) | 0.29 | 0.51 |
| Waist circumference | (per 10 cm increment) | 0.006 (-0.367 to 0.378) | 0.98 | 0.412 (-0.117 to 0.941) | 0.13 | 0.22 |
| Current smoking habits | (yes vs no) | 1.693 (-0.060 to 3.445) | 0.06 | 0.749 (-0.902 to 2.401) | 0.37 | 0.44 |
| Current alcohol drinking | (yes vs no) | -0.403 (-1.145 to 0.340) | 0.29 | 1.364 (0.223 to 2.504) | 0.02 | 0.01 |
| Handgrip strength, kg | (per 5 kg decrement) | 0.480 (0.062 to 0.897) | 0.02 | 0.382 (-0.015 to 0.779) | 0.06 | 0.72 |
| Regular exercise | (yes vs no) | -0.071 (-0.851 to 0.709) | 0.86 | 0.496 (-0.579 to 1.572) | 0.36 | 0.40 |
| Frequency of working under sunlight exposure | (per 1-category increase in questionnaire ^a)^) | 0.326 (-0.079 to 0.732) | 0.11 | 0.297 (-0.186 to 0.779) | 0.23 | 0.93 |
| Frequency of nighttime awakenings | (per 1-category increase in questionnaire ^a)^) | 0.131 (-0.215 to 0.478) | 0.46 | 0.453 (0.001 to 0.905) | 0.049 | 0.27 |
| Frequency of constipation or diarrhea | (per 1-category increase in questionnaire ^a)^) | -0.332 (-1.190 to 0.526) | 0.45 | 0.693 (-0.146 to 1.533) | 0.10 | 0.10 |
| Frequency of skin care | (per 1-category increase in questionnaire ^b)^) | -0.287 (-0.575 to 0.000) | 0.050 | 0.057 (-0.276 to 0.390) | 0.74 | 0.12 |
| Abbreviations: CI, confidence interval; HDL, high-density lipoprotein; AST, aspartate aminotransferase; ALT, alanine aminotransferase; γ-GTP, gamma-glutamyl transpeptidase.   1. Definition of category: 1, rarely; 2, several times a month; 3, several times a week; 4, almost every day. 2. Definition of category: 1, rarely; 2, approximately 1–2 days a week; 3, approximately 3–4 days a week; 4, approximately 5 days a week; 5, almost every day. 3. Difference between skin age and chronological age was calculated as the residual of a linear regression model with chronological age as the independent variable and skin age as the dependent variable. Values were adjusted for age. 4. Among 527 participants, 518 provided blood samples in a fasting state, and 9 had postprandial blood sampling. | | | | | | |

| **Supplementary Table 3. Association of lifestyle and cardiovascular risk factors with the difference between skin age and chronological age by sex, in the multivariable-adjusted model** | | | | | | | |
| --- | --- | --- | --- | --- | --- | --- | --- |
| Lifestyle and cardiovascular risk factors | | Unit | Multivariable-adjusted  (Full-model) | |  | Multivariable-adjusted  (Backward elimination)^d)^ | |
|  |  |  | Difference (95% CI) between skin age and chronological age, years ^c)^ | p value |  | Difference (95% CI) between skin age and chronological age, years ^c)^ | p value |
| **Women** | |  |  |  |  |  |  |
|  | Age | (per 10 years increment) | -0.368 (-0.951 to 0.216) | 0.22 |  | -0.391 (-0.929 to 0.146) | 0.15 |
|  | Systolic blood pressure | (per 10 mmHg increment) | 0.283 (0.017 to 0.549) | 0.04 |  | 0.272 (0.008 to 0.537) | 0.04 |
|  | Blood glucose ^e)^ | (per 1 mmol/L increment) | 0.595 (-0.079 to 1.270) | 0.08 |  | 0.704 (0.076 to 1.332) | 0.03 |
|  | Serum non-HDL cholesterol | (per 1 mmol/L increment) | -0.269 (-0.709 to 0.171) | 0.23 |  | ns |  |
|  | Serum HDL cholesterol | (per 1 mmol/L decrement) | 0.821 (-0.109 to 1.751) | 0.08 |  | ns |  |
|  | Serum uric acid | (per 100 µmol/L increment) | 0.362 (-0.279 to 1.003) | 0.27 |  | ns |  |
|  | Serum γ-GTP | (per 2-times increment) | 0.298 (-0.135 to 0.732) | 0.18 |  | ns |  |
|  | Waist circumference | (per 10 cm increment) | -0.354 (-0.796 to 0.088) | 0.12 |  | ns |  |
|  | Current smoking habits | (yes vs no) | 1.234 (-0.534 to 3.002) | 0.17 |  | 1.636 (-0.079 to 3.352) | 0.06 |
|  | Current alcohol drinking | (yes vs no) | -0.404 (-1.167 to 0.359) | 0.30 |  | ns |  |
|  | Handgrip strength, kg | (per 5 kg decrement) | 0.478 (0.055 to 0.902) | 0.03 |  | 0.535 (0.125 to 0.945) | 0.01 |
|  | Frequency of working under sunlight exposure | (per 1-category increase in questionnaire ^a)^) | 0.362 (-0.047 to 0.770) | 0.08 |  | 0.379 (-0.027 to 0.785) | 0.07 |
|  | Frequency of nighttime awakenings | (per 1-category increase in questionnaire ^a)^) | 0.084 (-0.262 to 0.431) | 0.63 |  | ns |  |
|  | Frequency of constipation or diarrhea | (per 1-category increase in questionnaire ^a)^) | -0.421 (-1.280 to 0.439) | 0.34 |  | ns |  |
|  | Frequency of skin care | (per 1-category increase in questionnaire ^b)^) | -0.168 (-0.460 to 0.123) | 0.26 |  | ns |  |
| **Men** | |  |  |  |  |  |  |
|  | Age | (per 10 years increment) | -0.506 (-1.285 to 0.274) | 0.20 |  | -0.391 (-1.125 to 0.344) | 0.29 |
|  | Systolic blood pressure | (per 10 mmHg increment) | 0.299 (-0.126 to 0.723) | 0.17 |  | 0.349 (-0.046 to 0.745) | 0.08 |
|  | Blood glucose ^e)^ | (per 1 mmol/L increment) | -0.024 (-0.614 to 0.565) | 0.94 |  | ns |  |
|  | Serum non-HDL cholesterol | (per 1 mmol/L increment) | 0.330 (-0.336 to 0.997) | 0.33 |  | ns |  |
|  | Serum HDL cholesterol | (per 1 mmol/L decrement) | -0.439 (-1.990 to 1.113) | 0.58 |  | ns |  |
|  | Serum uric acid | (per 100 µmol/L increment) | 0.131 (-0.609 to 0.870) | 0.73 |  | ns |  |
|  | Serum γ-GTP | (per 2-times increment) | 0.613 (0.015 to 1.212) | 0.04 |  | 0.732 (0.178 to 1.286) | 0.01 |
|  | Waist circumference | (per 10 cm increment) | 0.334 (-0.235 to 0.903) | 0.25 |  | ns |  |
|  | Current smoking habits | (yes vs no) | 0.245 (-1.398 to 1.888) | 0.77 |  | ns |  |
|  | Current alcohol drinking | (yes vs no) | 1.071 (-0.188 to 2.331) | 0.09 |  | 1.183 (0.023 to 2.343) | 0.046 |
|  | Handgrip strength, kg | (per 5 kg decrement) | 0.491 (0.091 to 0.891) | 0.02 |  | 0.485 (0.103 to 0.867) | 0.01 |
|  | Frequency of working under sunlight exposure | (per 1-category increase in questionnaire ^a)^) | 0.406 (-0.076 to 0.889) | 0.10 |  | 0.417 (-0.045 to 0.879) | 0.08 |
|  | Frequency of nighttime awakenings | (per 1-category increase in questionnaire ^a)^) | 0.258 (-0.204 to 0.719) | 0.27 |  | ns |  |
|  | Frequency of constipation or diarrhea | (per 1-category increase in questionnaire ^a)^) | 0.370 (-0.457 to 1.198) | 0.38 |  | ns |  |
|  | Frequency of skin care | (per 1-category increase in questionnaire ^b)^) | 0.208 (-0.121 to 0.537) | 0.21 |  | ns |  |
| Abbreviations: CI, confidence interval; HDL, high-density lipoprotein; γ-GTP, gamma-glutamyl transpeptidase; ns, not selected.   1. Definition of category: 1, rarely; 2, several times a month; 3, several times a week; 4, almost every day. 2. Definition of category: 1, rarely; 2, approximately 1–2 days a week; 3, approximately 3–4 days a week; 4, approximately 5 days a week; 5, almost every day. 3. Difference between skin age and chronological age was calculated as the residual of a linear regression model with chronological age as the independent variable and skin age as the dependent variable. 4. Variable selection was performed using a backward elimination procedure with a selection threshold of p < 0.10 to identify factors associated with the difference between skin age and chronological age, where age was included in the model irrespective of statistical significance. 5. Among 527 participants, 518 provided blood samples in a fasting state, and 9 had postprandial blood sampling. | | | | | | | |
